# Identification and characterization of gut-associated lactic acid bacteria isolated from the bean bug, *Riptortus pedestris* (Hemiptera: Alydidae)

**DOI:** 10.1371/journal.pone.0281121

**Published:** 2023-03-30

**Authors:** Okhee Choi, Yeyeong Lee, Byeongsam Kang, Su Kyung Cho, Yongsung Kang, Dong-Wan Kang, Seul-Bi Lee, Sung-Mun Bae, Jinwoo Kim

**Affiliations:** 1 Institute of Agriculture and Life Science, Gyeongsang National University, Jinju, Republic of Korea; 2 Department of Plant Medicine, Gyeongsang National University, Jinju, Republic of Korea; 3 Gyeongsangnam-do Agricultural Research and Extension Services, Jinju, Republic of Korea; University of South Dakota Sanford School of Medicine, UNITED STATES

## Abstract

Lactic acid bacteria (LAB) are beneficial bacteria for humans and animals. However, the characteristics and functions of LAB in insects remain unclear. Here, we isolated LAB from the gut of *Riptortus pedestris*, a pest that is a significant problem in soybean cultivation in Korea, and identified two *Lactococcus lactis* and one *Enterococcus faecalis* using matrix-associated laser desorption/ionization-time of flight and 16S rRNA analyses. All three LAB strains survived at pH 8, and *L*. *lactis* B103 and *E*. *faecalis* B105 survived at pH 9 for 24 h. In addition, these strains survived well in simulated gastric juice of humans containing pepsin and exhibited high resistance to bile salts. Two strains of *L*. *lactis* and one of *E*. *faecalis* maintained constant density (> 10^4^ colony-forming units [CFU]/mL) at pH 2.5, but viability at pH 2.2 was strain-dependent. The three LAB were reinoculated into second-instar nymphs of *R*. *pedestris* and colonized well, reaching a constant density (> 10^5^ CFU/gut) in the adult insect gut. Interestingly, feeding of these LAB increased the survival rate of insects compared to the negative control, with the largest increase seen for *L*. *lactis* B103. However, the LAB did not increase the weight or length of adult insects. These results indicate that insect-derived LAB possess the traits required for survival under gastrointestinal conditions and have beneficial effects on insect hosts. The LAB infection frequency of the wild bean bug populations was 89% (*n* = 18) in Gyeongsangnam-do, South Korea. These LAB can be utilized as a novel probiotic in the cultivation of beneficial insects. This study provides fundamental information about the symbiosis between insects and LAB, and a novel concept for pest control.

## Introduction

Insects are the most diverse group of animals in terms of species number and have the largest biomass in almost all environments on earth [1, 2]. The diversification of insects is closely related to the function of microbial communities inhabiting their intestinal systems [3–5]. These microorganisms promote the digestion of food in the gut, protect against predators and pathogens, enhance immune responses, and influence intraspecific communication and behavior [2, 5–7]. In this manner, insects have evolved while maintaining symbiotic relationships with microorganisms. Numerous studies have investigated host–microbe interactions in the context of intestinal microbiota using insects such as fruit flies, social bees, termites, and cockroaches [8–11].

Lactic acid bacteria (LAB) are beneficial microorganisms found in humans, insects, and animals [12]. LAB fermentation plays an important role in the food industry, particularly for dairy products. Strains of LAB are generally recognized as safe (GRAS) food-grade microorganisms and used as probiotics to benefit human health [13]. Among insect-derived LAB, symbiotic LAB of honeybees have been studied extensively [14–16]. Honeybees are essential pollinators in natural ecosystems and agricultural production. Their symbiotic LAB have been suggested to play a significant role in honeybee health and honey production. LAB from honeybees such as *Lactobacillus johnsonii*, *Lactobacillus plantarum*, *and Lactobacillus brevis* have shown inhibitory effects against pathogens such as *Paenibacillus larvae* and *Melissococcus plutonius* [16].

The bean bug *Riptortus pedestris* (Hemiptera: Alydidae) is an important pest that infests leguminous crops, fruit trees, and grains [17]. These insects occur widely in Southeast Asia, including Korea, Japan, and China [18, 19]. In Korea, the abundance of *R*. *pedestris* has increased rapidly since 2000, resulting in severe losses of leguminous crops [20, 21]. In *R*. *pedestris*, the nymphs of each generation acquire bacteria of the genus *Burkholderia* from the soil environment [22]. These bacteria are capable of highly specific and efficient colonization of the insect gut [23]. Recent studies have demonstrated that symbiotic *Burkholderia* (currently *Caballeronia*) have has beneficial effects, such as improving growth of the host and conferring resistance to pesticides on the host [22, 24, 25].

In this study, we focused on the isolation, identification, and characterization of insect-derived LAB. *R*. *pedestris*, a pest that has recently become problematic in Korea, was selected as a source for isolation of LAB. This insect is easy to collect and rear, and convenient to handle, making it an ideal experimental material. Here, we isolated and identified LAB associated with the gut of the bean bug and investigated their characteristics and functions. These insect-derived LAB are expected to be widely used as probiotics in the insect industry, including for the rearing of beneficial insects.

## Materials and methods

### Insect source and rearing

Adult bean bugs, *R*. *pedestris*, were collected from soybean fields in Jinju, South Korea. Permission for field site access is not required as the study carried minimal risk to the subjects. Bean bugs were reared in plastic containers (35 × 35 × 40 cm) with soybeans and distilled water containing 0.05% ascorbic acid (DWA) at 28°C under long-day conditions (16 h light, 8 h dark) [23]. Cotton pads were placed inside the plastic container to collect the insect eggs. Eggs were collected and transferred to new container for hatching. Newly hatched nymphs were reared to second-instar nymphs for the subsequent experiment.

### Isolation of LAB and culture conditions

Adult bean bugs, *R*. *pedestris*, were collected from soybean fields in Jinju, South Korea in 2018. Twenty *R*. *pedestris* adults were rinsed twice with sterile water and surface-sterilized with 70% ethanol, and their guts were carefully removed and collected in 10 mM phosphate buffer, pH 7.0 (PB). The guts were homogenized with a plastic pestle and serially diluted with PB. The diluted gut solution was spread onto de Man, Rogosa, and Sharpe (MRS) agar medium (Difco, Detroit, MI, USA) and incubated at 28°C for 3 days in a CO_2_ incubator (Thermo Scientific, Waltham, MA, USA). Bacterial colonies that were uniform, round, and white were selected, purified, and stored at -80°C in MRS broth mixed with glycerol (25%, v/v).

### Identification of LAB

To confirm the identity of bacterial isolates (B103–B105), whole-cell matrix-assisted laser desorption/ionization-time of flight (MALDI-TOF) mass spectrometry using the program MALDI Biotyper v.3.0 (Bruker Daltonics, Bremen, Germany) were performed according to the manufacturer’s instructions (Bruker Daltonics). Bacterial samples for MALDI-TOF MS were prepared as previously described [26]. Mass spectra were analyzed using a microflex LT mass spectrometer (Bruker Daltonics) and default parameters (positive linear mode; laser frequency, 60 Hz; ion source 1voltage, 20 kV; ion source 2voltage, 16.7 kV; lens voltage, 7.0 kV; and mass range, 2 kDa to 20 kDa). For each spectrum, 240 laser shots in 40-shot steps from different positions of the sample spot were accumulated and analyzed (automatic mode, default settings) as previously described [26]. MALDI-TOF MS data were interpreted according to the manufacturer’s instructions (Bruker Daltonics). Scores of ≥2.0 are considered reliable for the species level, and scores of ≥1.7 but <2.0 are acceptable for the genus level, and scores below 1.7 are considered unreliable [26].

The partial 16S rRNA region was amplified with the primers 27mF (5**′**-AGAGTTTGATCMTGGCTCAG-3′) and 1492mR (5**′**-GGYTACCTTGTTACGACTT-3**′**) [27]. Total DNA extraction was conducted as described by Choi et al. (2019) [28]. Polymerase chain reaction (PCR) was performed on a T100 Thermal Cycler (Bio-Rad, Hercules, CA, USA) using PCR premix (Bioneer, Daejeon, Korea), 10 ng genomic DNA, and 1 mM of each primer with a program of 98°C for 30 s, 55°C for 30 s, and 70°C for 1 min, followed by a final 4-min extension at 72°C. The PCR product was confirmed using 0.8% agarose gel electrophoresis and purified using the Expin Gel SV kit (GeneAll Biotechnology, Seoul, Korea). Sequencing was performed by Macrogen’s sequencing service (Macrogen, Daejeon, Korea). The resulting DNA sequences were analyzed using the National Center for Biotechnology Information GenBank database (https://www.ncbi.nlm.nih.gov/blast/). A phylogenetic tree based on the nucleotide sequences of the 16S rRNA gene was generated using MEGA 7.0 software and the neighbor-joining method [29]. *Streptococcus thermophilus* LMG 18311 was used as an outgroup because it is phylogenetically closely related to the genera *Lactococcus* and *Enterococcus* [30].

### Survival of LAB at alkaline pH

Survival of LAB strains at weak alkaline pH was tested. MRS broth was adjusted to pH 8 or 9 using 1 N NaOH. MRS broth adjusted to pH 7 was used as a control. Cells from 2-day-old cultures of the three isolates grown at 28°C were inoculated into pH-adjusted MRS broth at a concentration of approximately 10^9^ colony-forming units (CFU)/mL and incubated at 28°C for 24 h. Samples were taken at 6 h intervals, serially diluted, and spread onto MRS medium. The plates were incubated at 28°C for 3 days. The viable cell population was determined by colony counting.

### Survival of LAB in simulated gastric juice and bile salt solution of humans

Simulated gastric juice of humans was prepared by adding 1% pepsin to MRS broth adjusted to pH 2.2 or 2.5 using 1 N HCl, according to a previously reported method [31]. Cells from 2-day-old cultures of the three isolates grown at 28°C were inoculated into simulated gastric juices at a concentration of approximately 10^9^ colony-forming units (CFU)/mL and incubated at 28°C for 2 h. Samples were taken at 30 min intervals, serially diluted, and spread onto MRS medium. The plates were incubated at 28°C for 3 days. The viable cell population was determined by colony counting.

For the bile tolerance assay, cells from 2-day-old cultures of the three isolates grown at 28°C were inoculated into MRS broth containing 0.1 or 0.5% (w/v) bile salt (oxgall) (Difco) at a concentration of approximately 10^9^ CFU/mL and then incubated at 28°C for 24 h according to a previously reported method [32]. Samples were taken at 6 h intervals, serially diluted, and spread onto MRS medium. The plates were incubated at 28°C for 3 days. The viable cell population was determined through colony counting.

### Determination of glucose utilization and pH values

To assess the glucose utilization of the three isolates, a D-glucose assay kit (glucose oxidase/peroxidase [GOPOD]; Megazyme, Wicklow, Ireland) was used according to the manufacturer’s instructions [33]. The three isolates were grown on MRS broth at 28°C for 3 days and sampled at 12-h intervals; the supernatants were collected. Then, 3 mL GOPOD reagent was added to each 100-μL aliquot of supernatant and incubated at 42°C for 20 min. Distilled water was used as the reagent blank solution. After the color reaction, absorbance of the samples was read at 510 nm using a spectrophotometer (Genesys 6; Thermo Electron Corp., Waltham, MA, USA). The pH of the MRS culture medium of the isolates was measured at 4-h or 24-h intervals for 3 days using a pH meter (SevenEasy; Mettler-Toledo, Greifensee, Switzerland).

### Analysis of organic acids

To determine whether the three isolates use glucose as a carbon source for conversion to lactic acid, the isolates were cultured in MRS broth containing 2% glucose at 28°C for 2 days. The bacterial cultures were centrifuged and the supernatant were collected. Each supernatant was filtered through a 0.45-μm cellulose acetate filter for high-performance liquid chromatography (HPLC) analysis. The concentrations of organic acids, such as lactic acid and acetic acid, were determined through HPLC (L-2200; Hitachi, Tokyo, Japan) using an ultraviolet detector (L-2400; Hitachi) and a column (Metacarb 87H; Varian, Palo Alto, CA, USA). HPLC profiles of the culture filtrates were analyzed through comparison with the elution profiles of standard organic acids injected separately.

### Bean bug feeding and evaluation of LAB survival in the gut

A bean bug feeding trial was conducted to investigate the survival of LAB in the gut. Rifampicin, a bacterial RNA polymerase inhibitor, was previously used for bacterial colonization monitoring studies in bean bugs [34]. Spontaneous rifampicin-resistant strains of LAB were obtained through sequential selection on MRS agar containing rifampicin starting at 5 μg/mL and up to 25 μg/mL. Spontaneous rifampicin-resistant strains of LAB were cultured on MRS containing 25 μg/mL rifampicin at 28°C for 2 days, and the cultured cells were harvested, washed with DWA, and suspended in DWA at a concentration of approximately 10^8^ CFU/mL. Newly molted second-instar nymphs were not supplied with DWA for 24 h, and the thirsty nymphs were then supplied with DWA containing LAB cells for 24 h. After inoculation, the nymphs were reared as described above. At 21 days post-inoculation, five adult bean bugs were dissected and their guts were transferred to a tube containing 500 μL PB. The guts were completely homogenized with a plastic pestle and serially diluted with PB. The diluted solution was spread onto MRS agar containing 25 μg/mL rifampicin. After 3 days of incubation at 28°C, colonies on the plates were counted.

### Evaluation of *R*. *pedestris* survival rate, weight, and length

To assess the survival rate of insects after feeding of LAB cells to second instar nymphs, dead insects were counted daily for 20 days until the nymphs became adults. At 21 days after bacterial inoculation, adults of *R*. *pedestris* were immersed in acetone for 5 min, and completely dried at 70°C in an oven; their dry body weight was then measured. The insects were not fed for 24 h before killing. Body length was measured from the head to the tip of the abdomen. Mean differences are shown on Cumming estimation plots [35] as dots; 95% confidence intervals are indicated by the ends of the vertical bars. All estimation plots were generated using https://www.estimationstats.com/#/.

### Natural LAB infection in the wild population of bean bug

To determine the natural LAB infection rate of wild bean bug populations, *R*. *pedestris* adults were collected from soybean fields located in 18 regions of Gyeongsangnam-do, South Korea during 2021–2022 (one per region). LAB isolation was performed according to the method described above. Among the colonies formed on MRS after culturing in a CO_2_ incubator, the identical colonies having the same color and shape as *L*. *lactis* and *E*, *faecalis* were purely isolated and identified using the MALDI-TOF MS utility.

## Results

### Identification of LAB in the gut of *Riptortus pedestris*

To isolate the LAB associated with the bean bug gut, bacteria were isolated from the guts of healthy adult *R*. *pedestris*. Gram-positive, oxidase-negative bacteria with round, white colonies were consistently recovered on MRS medium containing 2% glucose. Three bacterial isolates (labeled B103–B105) were selected for identification and characterization.

MALDI-TOF mass spectrometric analysis revealed that B103 and B104 were *Lactococcus lactis* with scores of 2.236 and 2.256 (score ≥2.0 = identification to the species level [26]), respectively, while B105 yielded score of 2.331 as *Enterococcus faecalis*.

**Fig 1:**
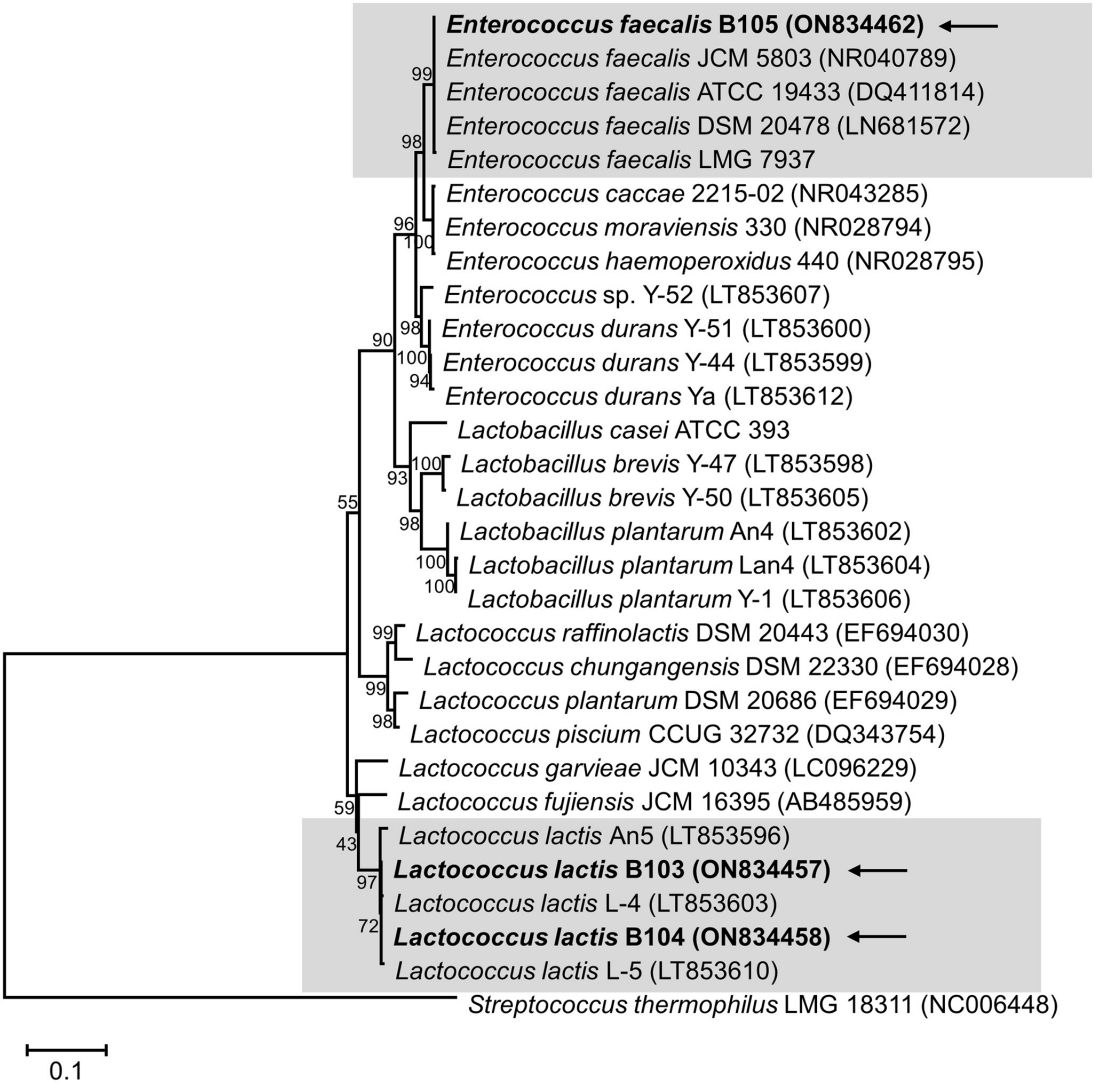
Phylogenetic tree of LAB based on 16S rRNA gene sequences constructed using the neighbor-joining method. The numbers above the branches are bootstrap values. Bars indicate the number of nucleotide substitutions per site. Isolates used in this study are indicated in bold and with arrows. *Streptococcus thermophilus* LMG 18311 (NC006448) was used as the outgroup.

16S rRNA gene sequence analysis revealed that two isolates (B103 and B104) had identical 16S rRNA gene sequences. BLAST search confirmed that the 16S rRNA gene sequences of B103 and B104 (1,406 bp; GenBank accession nos. ON834457 and ON834458) shared 100% similarity to *Lactococcus lactis* 1700 (MT597572) isolated in China, and the 16S rRNA gene sequence of B105 (1,401 bp; GenBank accession no. ON834462) shared 100% similarity to *Enterococcus faecalis* ABCW (ON564563) obtained from cheese whey in India. In phylogenetic analysis, B103 and B104 clustered in the same group as *Lactococcus lactis*, while B105 clustered in the same group as *Enterococcus faecalis* (Fig 1).

### Survival of LAB at weak alkaline pH

**Fig 2:**
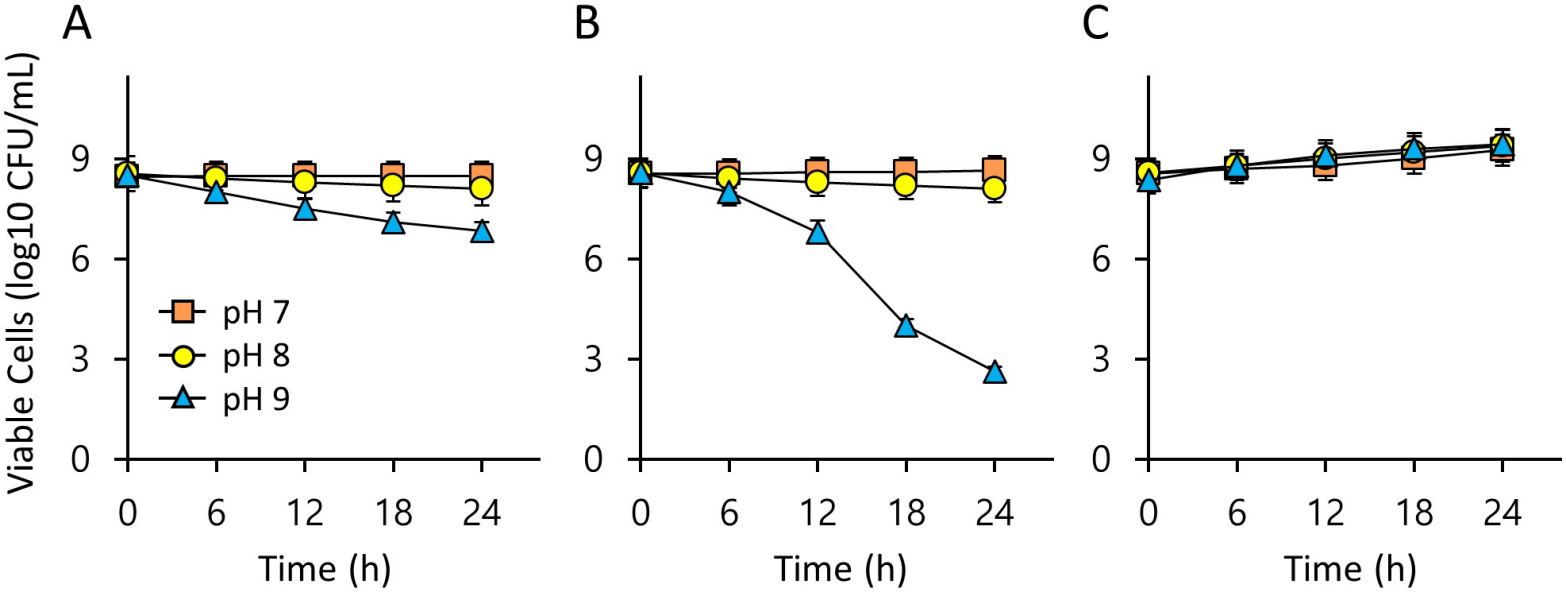
Survival of LAB at weak alkaline pH. Viable cells of *L*. *lactis* B103 (A), *L*. *lactis* B104 (B), and *E*. *faecalis* B105 (C) over time in MRS broth at pH 7, pH 8, or pH 9. Values are means of data from triplicate experiments each of which contained three technical replicates, with the standard deviation (SD) indicated by vertical bars.

In most insects, the digestive juices in the midgut generally fall within the pH range of 6–8 [36–38]. To evaluate the survival of LAB under weak alkaline conditions, we assessed the survival of three LAB strains in MRS broth at pH 8 and 9 for 24 h. The viable cell counts of the three LAB strains at pH 7, used as a control, did not change over 24 h, and were similar even at pH 8 (Fig 2). *L*. *lactis* B103 decreased slowly by 6.83 log10 CFU/mL over 24 h of exposure to pH 9 (Fig 2A), while the survival of *L*. *lactis* B104 decreased sharply after 24 h of exposure to pH 9, down to 2.63 log10 CFU/mL (Fig 2B). These results indicate that all tested strains are resistant to the simulated insect gut pH environment and that species specificity appears among LAB strains even within a species.

### Survival of LAB in simulated gastric juice and bile salt solution of humans

**Fig 3:**
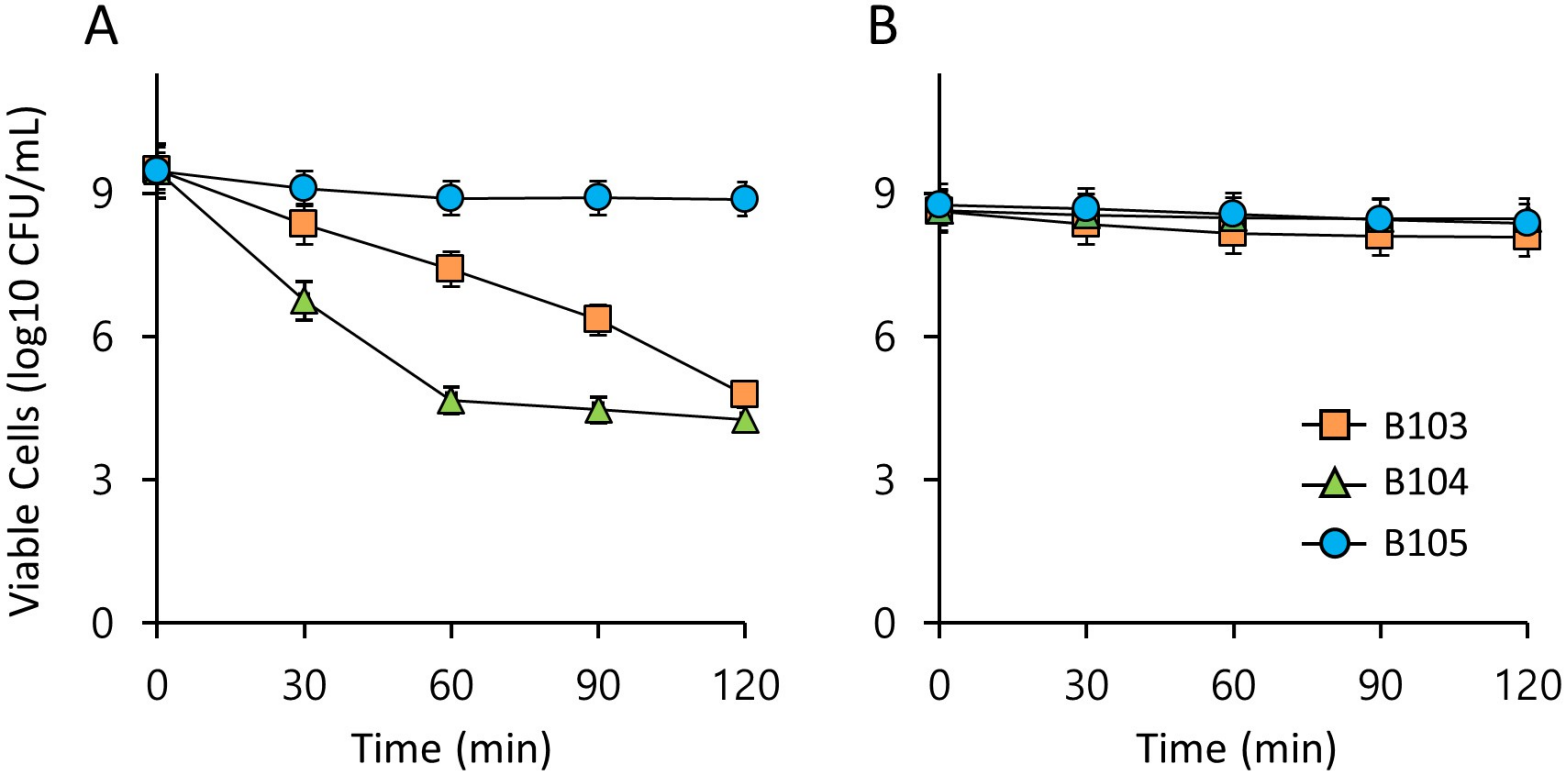
Survival of LAB in simulated gastric juice of humans. Viable cells of LAB over time in MRS broth containing 1% pepsin at pH 2.2 (A) or pH 2.5 (B). Values are means of data from triplicate experiments, with the standard deviation (SD) indicated by vertical bars.

We next assessed the viability of LAB in a low pH environment simulating the human gut to determine critical probiotic availability. To evaluate the survival of LAB under acidic conditions, we assessed the survival of three LAB strains in simulated gastric juice (pH 2.2 or 2.5) containing 1% pepsin for 120 min. *E*. *faecalis* B105 exhibited the highest survival rate over 120 min at pH 2.2, while the survival of *L*. *lactis* B104 decreased sharply after 60 min of exposure, with a reduction of 4.26 log10 CFU/mL seen at 120 min. B103, a second *L*. *lactis* strain, decreased slowly, by 4.78 log10 CFU/mL over 120 min (Fig 3A). These results indicate that the survival rate differs among LAB strains, even within a species. *L*. *lactis* B103, *L*. *lactis* B104, and *E*. *faecalis* B105 all had high survival rates at pH 2.5 over 120 min of exposure (Fig 3B). These results indicate that all tested strains exhibit resistance to the gastric juice environment.

**Fig 4:**
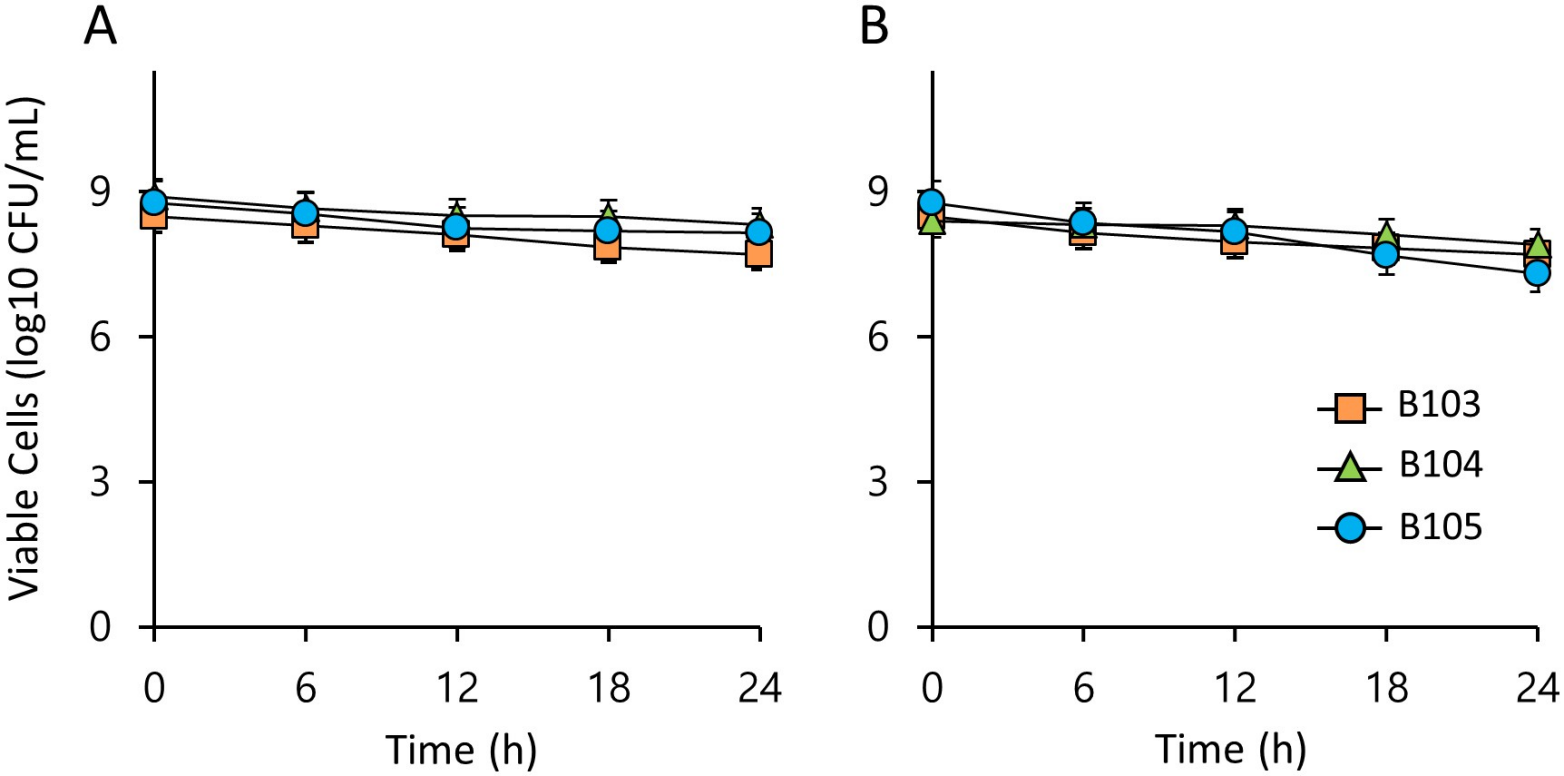
Survival of LAB in simulated bile juice of humans. Viable cells of LAB over time in MRS broth containing 0.1% (w/v) (A) or 0.5% (w/v) (B) bile salt. Values are means of data from triplicate experiments, with the SD indicated by vertical bars.

To evaluate whether bile salts reduce the survival of LAB, the three LAB strains were assessed for survival in MRS media containing 0.1% and 0.5% bile salt (oxgall). All tested LAB isolates showed high survival rates after 24 h of exposure to 0.1% and 0.5% oxgall (Fig 4A and 4B). These results indicate that the three LAB strains have significant tolerance to bile salts.

### Glucose utilization of LAB and pH change

**Fig 5:**
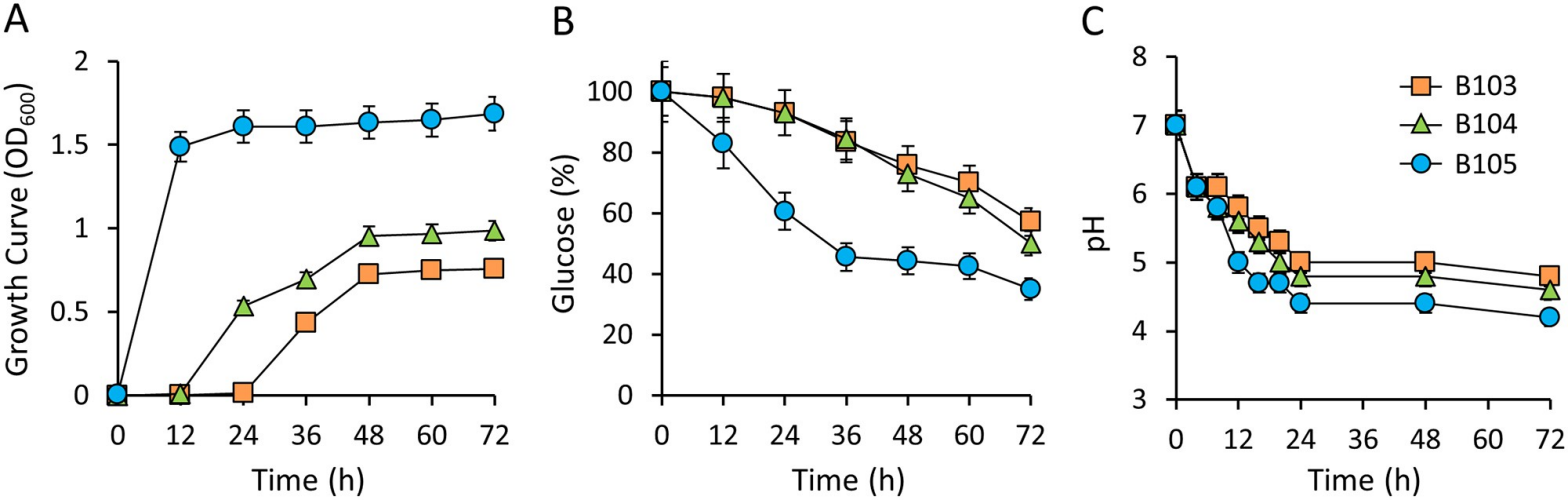
Effects of glucose utilization by LAB. Growth curve (A), glucose utilization (B), and pH-change (C) in MRS medium. Values are means of data from triplicate experiments each of which contained three technical replicates, with the SD indicated by vertical bars.

To investigate glucose utilization by LAB and the associated pH change, glucose consumption of the three LAB strains was measured, as well as the pH of MRS medium. *E*. *faecalis* B105 grew rapidly, reaching the early stationary phase after 12 h in MRS medium, and its cell density led to an optical density at 600 nm (OD_600_) value of 1.69 after 72 h. In contrast, *L*. *lactis* B104 grew slowly and reached a low cell density of OD_600_ = 0.99 after 72 h, while B103, which is the same species as M104, had the lowest cell density of OD_600_ = 0.76 after 72 h (Fig 5A). *E*. *faecalis* B105 consumed glucose rapidly and had consumed 65% of the supplied glucose over 72 h, and its culture pH decreased rapidly to 4.4 over 24 h. In contrast, *L*. *lactis* B103 and B104 consumed 43% and 50% of the available glucose over 72 h, and reached pH of 5.0 and 4.8 over 24 h, respectively (Fig 5B and 5C). These results indicate that *E*. *faecalis* B105 utilizes glucose well and decreases pH more effectively than the other two isolates, consistent with the growth rates observed in MRS.

### Organic acid production by LAB from glucose

**Fig 6:**
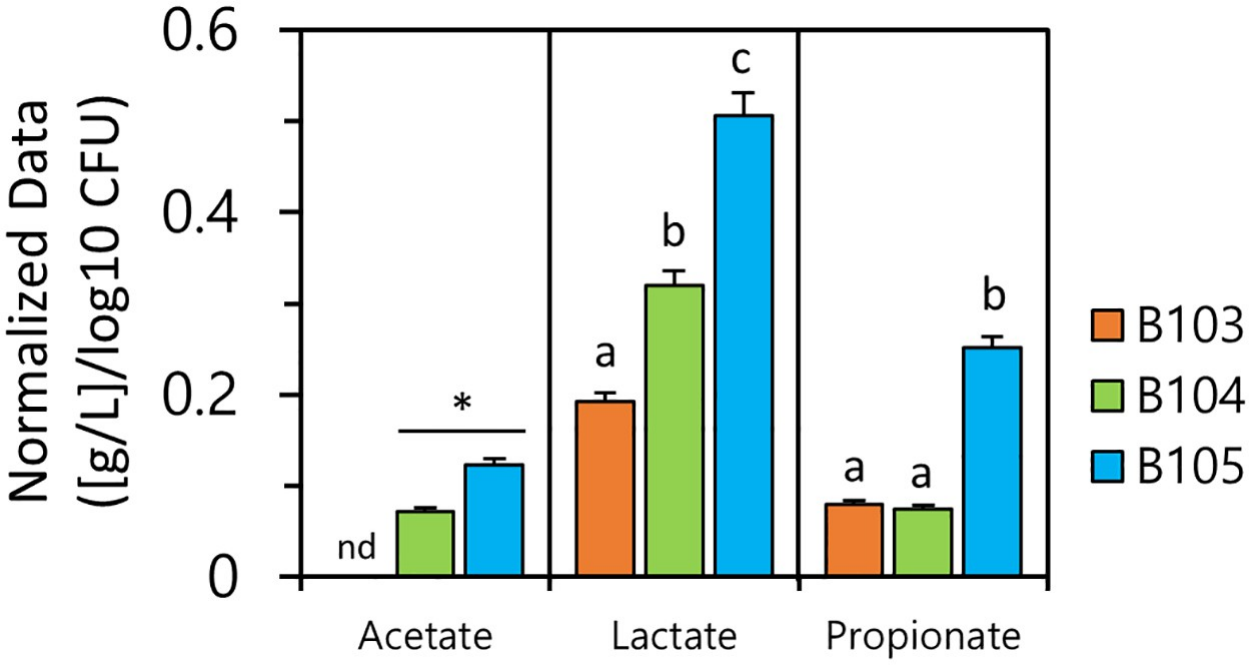
Organic acids production by LAB strains. Values for organic acid are means of data from triplicate experiments each of which contained three technical replicates. For presentation, all data were normalized to cell population (log10 CFU/mL). Columns marked with an asterisk (*) indicate statistically significant differences for acetate production between B104 and B105 strains using Student’s *t* test (*P* ≤ 0.05). nd: not detected. One-way ANOVA, Tukey test, *P* < 0.05 for lactate and propionate of the three LAB strains. Different letters indicate significant differences (*P* < 0.05) between LAB strains.

Organic acid production by LAB strains was investigated in MRS medium containing 20 g/L of glucose over 48 h. As shown in Fig 6, *E*. *faecalis* B105 exhibited a significant difference compared to the other strains, achieving higher contents of acetate, lactate, and propionate. *L*. *lactis* strain B104 produced significantly less acetate, lactate, and propionate than B105, but significantly more acetate and lactate than B103 strain. In contrast, B103 produced low contents of lactate and propionate; no acetate was detected. These results indicate that *E*. *faecalis* B105 efficiently utilized glucose for organic acid production. Organic acid production from glucose, differed among strains, even within the species *L*. *lactis*.

### Colonization of *R*. *pedestris* gut by LAB

**Fig 7:**
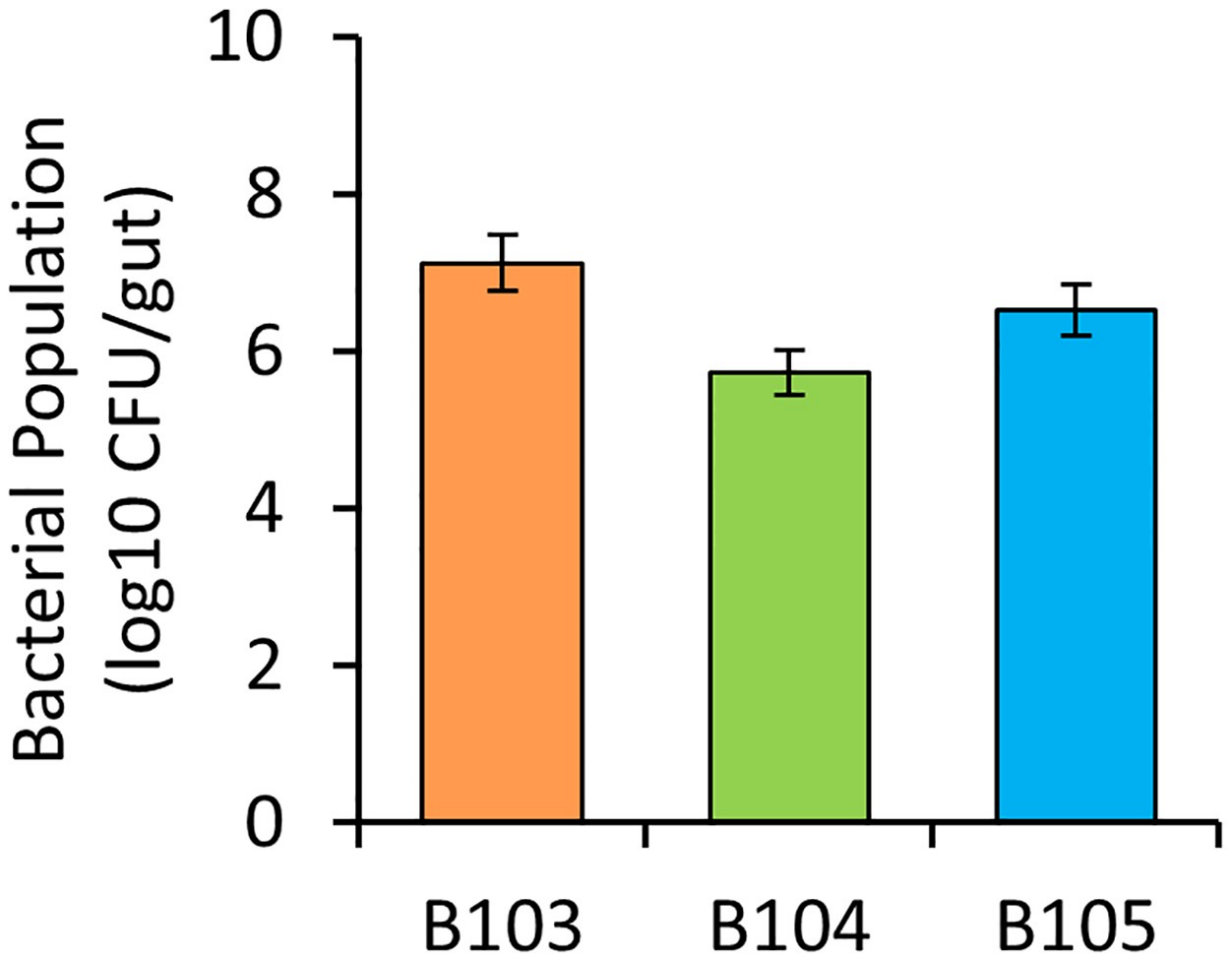
Colonizing populations of LAB in the guts of adult bean bugs. Second-instar nymphs were experimentally inoculated with LAB via feeding. At 21 days post-inoculation, adult bean bugs (*n* = 5) were used for colony population monitoring. This experiment was performed twice each of which contained three technical replicates. Error bars indicate SD.

To confirm whether the inoculated LAB efficiently colonizes the gut of *R*. *pedestris*, the number of bacteria per gut of adult insects that were inoculated at second-instar nymphs was investigated through bacterial colony counting. The bacterial population abundances of *L*. *lactis* B103 and B104, and *E*. *faecalis* B105 were 1.21 × 10^7^, 5.27 × 10^5^, and 3.24 × 10^6^ CFU/gut, respectively (Fig 7). These results indicate that the three tested LAB strains proliferated and successfully colonized the gut of insects to a consistent density after inoculation via feeding.

### Effect of LAB on the survival and growth of *R*. *pedestris*

**Fig 8:**
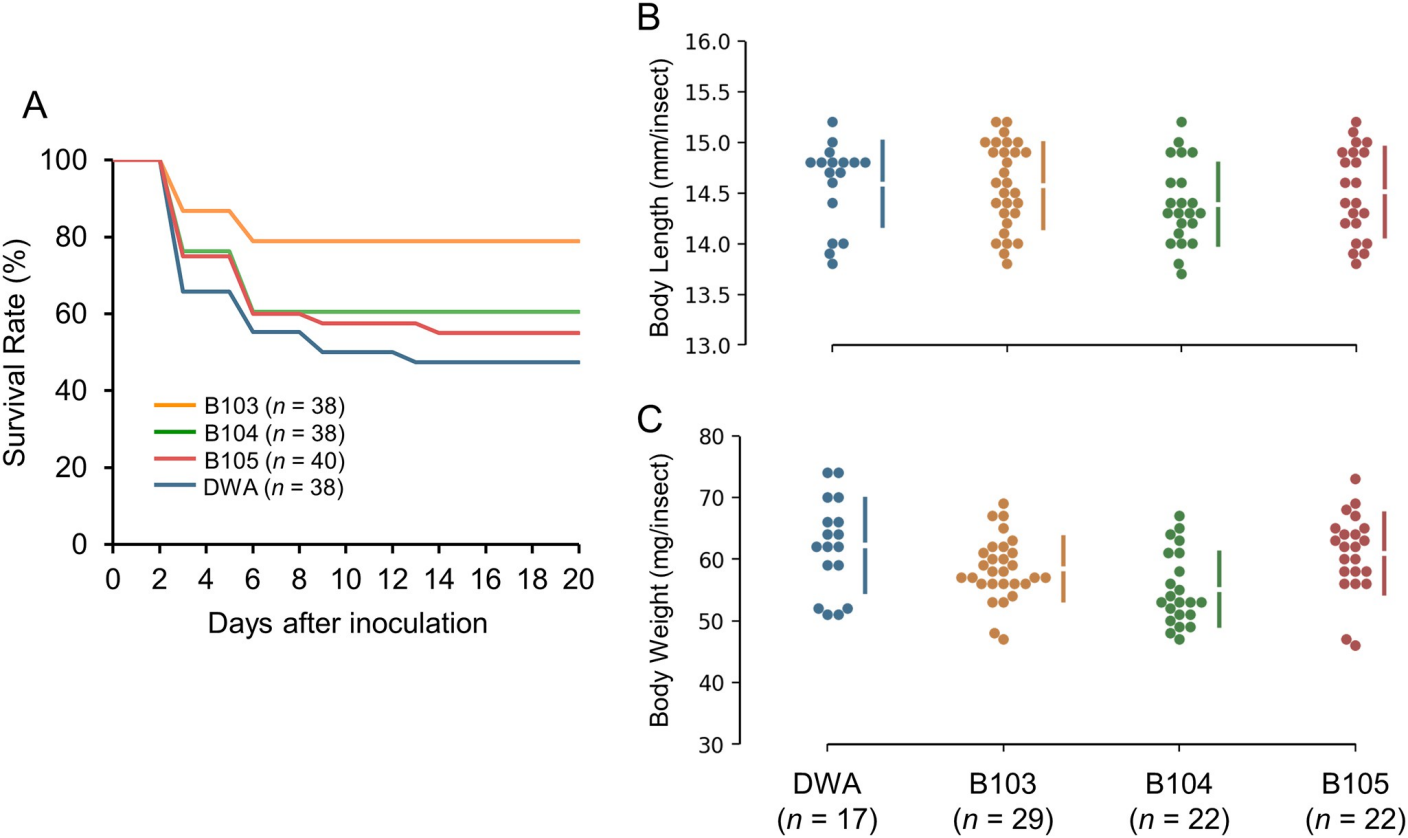
Effects of LAB on host insect development and growth. Improvement in the molt survival rate of bean bugs after feeding of LAB (A). Body length (B) and body weight (C) of adult insects after feeding of LAB. Second-instar nymphs were experimentally inoculated with LAB via feeding. Numbers of insects used in the experiments are indicated in parentheses. This experiment was performed once.

Next, to determine the impacts of the three LAB strains on the survival and growth of *R*. *pedestris*, the survival rate, length, and weight of experimentally inoculated adult insects were measured. The survival percentages of insects inoculated with *L*. *lactis* B103 and B104, and *E*. *faecalis* B105 were 78.9, 60.5, and 55%, respectively (Fig 8A). In contrast, the DWA treatment showed a survival rate of 47.3%. However, insects inoculated with the three LAB strains showed no difference in body weight or length compared to the DWA treatment (Fig 8B and 8C). These results indicate that the three LAB strains improve the viability of insects but have no growth-promoting effect.

### Natural LAB infection in the wild population of bean bug

**Fig 9:**
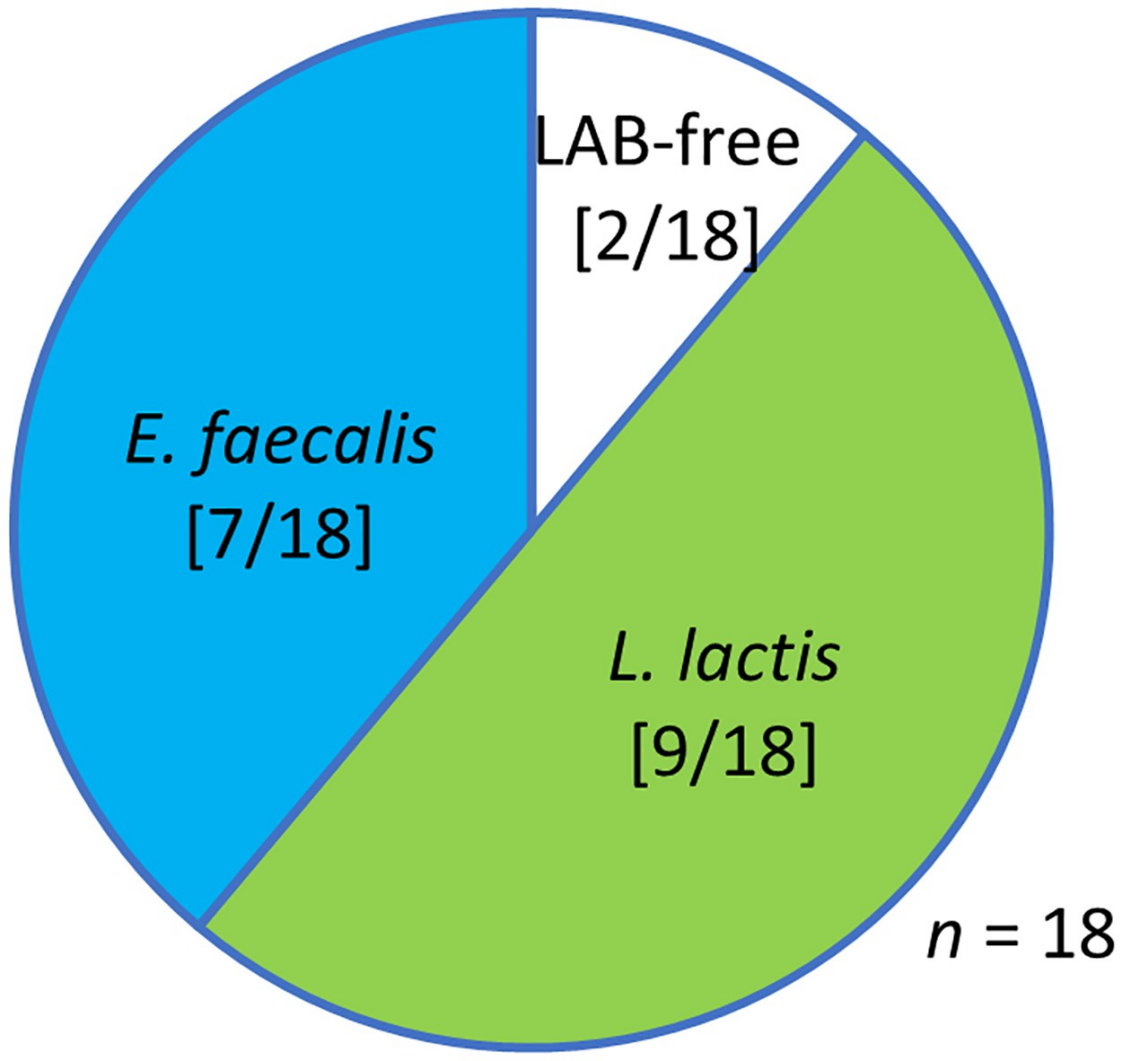
Discovery of LAB infection in *R*. *pedestris* in South Korea. Infection frequencies of LAB in the wild populations of *R*. *pedestris* in South Korea. The number of infected insects per number of all insects examined is shown in brackets.

LAB were isolated according to the method described above and identified using MALDI-TOF MS identification (score ≥2.0). The LAB infection frequency of the wild bean bug populations was 89% (16/18) (Fig 9). Two cultivable LAB, *L*. *lactis* and *E*. *faecalis*, were isolated from a single bean bug, but not simultaneously (Fig 9). These results indicate that the LAB–bean bug symbiosis depends on the habitat environment. The 16 LAB isolates obtained from the guts of bean bugs and their collection regions are listed in Table 1.

**Table 1:** LAB isolates obtained from the guts of bean bug adults collected from soybean fields during 2021–2022 in a natural infection rate survey.

Isolate	Species^a^	Region
LAB1	*Lactococcus lactis*	Hadong
LAB2	*Lactococcus lactis*	Namhae
LAB4	*Lactococcus lactis*	Sancheong
LAB5	*Lactococcus lactis*	Hamyang
LAB6	*Lactococcus lactis*	Gimhae
LAB7	*Enterococcus faecalis*	Yangsan
LAB8	*Lactococcus lactis*	Goseong
LAB9	*Lactococcus lactis*	Goeje
LAB10	*Lactococcus lactis*	Tongyeong
LAB12	*Enterococcus faecalis*	Hapcheon
LAB14	*Enterococcus faecalis*	Jinju
LAB16	*Enterococcus faecalis*	Sacheon
LAB17	*Enterococcus faecalis*	Uiryeong
LAB19	*Enterococcus faecalis*	Haman
LAB22	*Lactococcus lactis*	Milyang
LAB24	*Enterococcus faecalis*	Changynyeong

^a^Bacterial species were identified using MALDI-TOF MS (score ≥2.0).

## Discussion

The bean bug, *R*. *pedestris*, a problematic pest in soybean cultivation fields, was easily collected, and LAB were isolated from their guts. However, few species of bacteria were isolated, and the number of LAB was low. We isolated two strains of *Lactococcus lactis* and one of *Enterococcus faecalis* (Fig 1). The pH condition of the insect gut is somewhat alkaline, which may limit the growth and abundance of LAB [2]. In addition, the results impacted by LAB comprising only a small proportion of the total microflora [39, 40].

*L*. *lactis* is mainly isolated from plants and the environment. This LAB is used for cheese production and fermentation of foods including vegetables, meat, and wine [41, 42]. In addition, this bacterium has been genetically modified for the treatment of human diseases [43]. *L*. *lactis* has GRAS status and is beneficial to humans [44]. *L*. *lactis* has been isolated as a symbiotic bacterium from the oriental fruit fly and reported to affect the development, morphology, and survival of insects [45]. *L*. *lactis* was identified in mealworms (*Tenebrio molitor*) by 16S amplicon sequencing [46]. The LAB *E*. *faecalis* is isolated mainly from the guts of healthy humans and many animals. However, this species has also been reported as an opportunistic pathogen that produces bacteriocins and is highly resistant to antibiotics [47–49]. *E*. *faecalis* has been isolated from the feces of houseflies and cockroaches and reported as a symbiotic bacterium in the gut of the African cotton leafworm (*Spodoptera littoralis*) [50–52]. *E*. *faecalis* was also identified in mealworms (*Tenebrio monitor*) via 16S amplicon sequencing [46]. Some strains of this species are pathogenic to the larvae of the greater wax moth (*Galleia mellonella*) and African cotton leafworm (*S*. *littoralis*) [49, 51].

We focused on whether B103–B105 isolated from the bean bug can function as LAB. LAB benefit host cells only if they survive low pH conditions (pH 2.0–2.5) and toxic bile salts during passage through the gastrointestinal tract and adhere well to intestinal cells [52, 53]. The three isolates investigated in this study survived at densities of > 4.2 log10 CFU/mL in simulated gastric juice at pH 2.2 (Fig 3A). B103 and B104 belong to the same species of *L*. *lactis*, but showed different sensitivities to low pH. These results were consistent with previous research, which indicates that strains of the same *Lactobacillus* species may exhibit differences in viability at low pH and strain-dependent survival [52, 54]. All three isolates survived well in simulated gastric juice at pH 2.5 or 0.5% bile salt solution (Figs 3B and 4). All isolates produced organic acids, including lactic acid using glucose, and could lower the surrounding pH (Fig 6). Therefore, we suggest that these three isolates obtained from the gut of the bean bug exhibit the critical characteristics of probiotics. According to the viability assay in the simulated insect gut pH, i.e. weakly alkaline environment, all three strains survived well in MRS broth at pH 8, and *L*. *lactis* B103 and *E*. *faecalis* B105 survived at pH 9 except for *L*. *lactis* B104 (Fig 2). Colonization ability in the intestine is another important trait of LAB. B103–105 were confirmed to colonize the gut of adult insects at densities 10^5^–10^7^ CFU/gut (Fig 7). These results indicate that bacteria fed to second-instar nymphs proliferated and successfully colonized the gut, while the larvae molted into adults. In a previous study, the *Caballeronia* symbiont explicitly localized in the midgut crypts (M4) was monitored for localization using fluorescence labeling techniques [55]. In this study, we showed the total colony population of LAB instead of LAB localization in the gut of bean bugs, as it is currently unknown whether LAB are restricted to specific gut sections. Interestingly, these follow-up studies on this issue will have implications for understanding the natural ecology of LAB and will provide new opportunities for industrial strain development.

The *Caballeronia* symbiont has been reported as a specific symbiotic bacterium of bean bugs that colonizes the gut at high density, increases viability during the molting process and improves the growth of adults [56, 57]. In this study, inoculation with LAB also improved the viability of insects. However, no effect was observed on the length or weight of the insects (Fig 8). These results suggest that the three LAB strains tested here colonize the gut to a constant density and improve the detrimental physiological or biochemical alterations that occur during molting, allowing the host to survive well during the initial molting process. Additionally, these strains may benefit the host by supplying nutrients, such as organic acids and vitamins produced during fermentation. Bacteria coexisting with various insects, such as the fruit fly, African cotton stainer, beetle, and stinkbug, play beneficial roles in processes such as nutrition acquisition, vitamin supply, immunity, detoxification, and the acquisition of insecticide resistance [11, 58–60]. However, the symbiotic LAB–bean bug relationship is not host-specific as in the *Caballeronia*–bean bug symbiosis. The LAB species, including *L*. *lactis* and *E*. *faecalis*, have already been discovered in other insects. Depending on the insect species, their roles in beneficial or harmful relationships have been studied differently [44–51]. Moreover, LAB constitute a very small portion of the total microflora of insects [39, 40]. The regional differences in LAB infection found in the wild populations of bean bugs support that the symbiosis between *L*. *lactis* or *E*. *faecalis* and bean bug is not host-specific as in the *Caballeronia*–bean bug symbiosis but depends on the habitat environment. In addition, if methods such as fluorescent protein tagging or fluorescent in situ hybridization are applied to visualization LAB colonies in the insect gut, it will be helpful to understand the symbiosis of LAB-bean bug and will be necessary for follow-up studies.

The midgut of *R*. *pedestris* consists of four sections: M1, M2, M3, and M4, and the *Caballeronia* symbiont specifically colonizes the M4 section. Itoh et al. [34] demonstrated that in a single inoculation with taxonomically distinct bacteria, *Burkholderia fungorum* and *Pandoraea norimbergensis* colonized M4; however, other tested species, including *Enterococcus hirae* and *Lactobacillus casei*, showed little or no infection in M4. In co-infection, the symbiotic relationship between bean bugs and the *Caballeronia* symbiont is achieved by competition between gut bacteria in M4, and the *Caballeronia* symbiont consistently outperformed *B*. *fungorum* and *P*. *norimbergensis* in M4. It is currently unknown whether the LAB in this study will colonize the M4 section when co-infected with the *Caballeronia* symbiont, but results from previous reports suggest that it is unlikely that the LAB will colonize the M4 section. However, the results of LAB colonization in the guts and improvement of survival rate suggest a beneficial effect on insects. Further research is needed as the results on this issue will be valuable for future probiotics applications.

In this study, we isolated and identified *L*. *lactis* and *E*. *faecalis* from the bean bug *R*. *pedestris*, pest that causes severe damage to legumes in Korea, and demonstrated that these bacteria have the essential features of LAB. The present study is the first to report LAB isolated from the bean bug with potential application as probiotics. This study provides fundamental information about the symbiosis between insects and LAB, and a novel concept for pest control.
